# Double-Deck Metal Solenoids 3D Integrated in Silicon Wafer for Kinetic Energy Harvester

**DOI:** 10.3390/mi12010074

**Published:** 2021-01-12

**Authors:** Nianying Wang, Ruofeng Han, Changnan Chen, Jiebin Gu, Xinxin Li

**Affiliations:** 1 State Key Laboratory of Transducer Technology, Shanghai Institute of Microsystem and Information Technology, Chinese Academy of Sciences, Shanghai 200050, China; wangny@mail.sim.ac.cn (N.W.); hanruofeng@mail.sim.ac.cn (R.H.); ccn@mail.sim.ac.cn (C.C.); j.gu@mail.sim.ac.cn (J.G.); 2 School of Information Science and Technology, ShanghaiTech University, Shanghai 201210, China; 3 Chinese Academy of Sciences, Beijing 100049, China

**Keywords:** kinetic energy harvester, electromagnetic electricity generation, double-deck 3D metal solenoids, MEMS technology, micro-casting technology, wafer-level fabrication

## Abstract

A silicon-chip based double-deck three-dimensional (3D) solenoidal electromagnetic (EM) kinetic energy harvester is developed to convert low-frequency (<100 Hz) vibrational energy into electricity with high efficiency. With wafer-level micro electro mechanical systems (MEMS) fabrication to form a metal casting mold and the following casting technique to rapidly (within minutes) fill molten ZnAl alloy into the pre-micromachined silicon mold, the 300-turn solenoid coils (150 turns for either inner solenoid or outer solenoid) are fabricated in silicon wafers for saw dicing into chips. A cylindrical permanent magnet is inserted into a pre-etched channel for sliding upon external vibration, which is surrounded by the solenoids. The size of the harvester chip is as small as 10.58 mm × 2.06 mm × 2.55 mm. The internal resistance of the solenoids is about 17.9 Ω. The maximum peak-to-peak voltage and average power output are measured as 120.4 mV and 43.7 μW. The EM energy harvester shows great improvement in power density, which is 786 $\mu W/{\mathrm{cm}}^{3}$ and the normalized power density is 98.3 $\mu W/{\mathrm{cm}}^{3}/g$. The EM energy harvester is verified by experiment to be able to generate electricity through various human body movements of walking, running and jumping. The wafer-level fabricated chip-style solenoidal EM harvesters are advantageous in uniform performance, small size and volume applications.

## 1. Introduction

Great efforts have been made to develop small-size and low-power electronic microsystems for wearable applications [1,2], wireless sensors [3,4], wireless security-monitoring microsystems [5], etc. Miniaturization, low costs and low power consumption are the development tendency in the electronic microsystems. For many microsystems for filed applications where wired alternating current (AC) electric supply is not available and battery supply is often troublesome to periodically replace or recharge, on-site self-powered supply has become a major concern. Harvesting energy from the environment and then converting it into electrical power is a promising alternative to conventional power sources. Various ambient energy sources existing in the environment, such as vibration energy, solar energy, thermal energy and so on [6] have been tried for conversion into electrical power. Among these environmental energy sources, vibration energy has great advantage in broad accessibility, which is abundant in the environment and distributed in a wide frequency range, especially in low frequencies [7]. Thus, ambient vibration energy has attracted great attention in recent years. As one of the self-powered solutions of electronic micro-systems, various kinetic energy harvesters are explored extensively to convert ambient vibration energy to electricity.

Energy harvesters can be categorized by a generating mechanism such as a piezoelectric (PE) energy harvester [8], piezo-electromagnetic (PEM) hybrid energy harvester [9], electrostatic (ES) energy harvester [10,11], electromagnetic (EM) energy harvester [12,13,14,15], thermoelectric generator (TEG) [16], triboelectric nanogenerator (TENG) [17], pyroelectric generator [18,19], thermal radiation pyroelectric generator (PyG) [20,21], etc. Among these energy harvesters, EM energy harvesters can easily convert relative motion between a metal coil and magnet into electronic energy, and the output power density is relatively high. Unfortunately, conventional wire-wound EM energy harvesters suffer non-batch fabrication and large size, which obstructs miniaturization and highly uniform fabrication of EM energy harvesters and chip-style integration in microsystems.

This paper proposes double-deck three-dimensional (3D) metal solenoids that are fabricated with a silicon-wafer based integration method and can generate much larger power density than single-layer solenoid structure [13]. A silicon mold for the 3D metal solenoid with double-deck metal coils is firstly fabricated by micro electro mechanical systems (MEMS) techniques including through-wafer deep reactive ion etch (DRIE). Then, the pre-fabricated silicon molds, which consist of six-layer silicon wafers are stacked together for molten alloy filling (or say casting). With the wafer-level micro-casting technique [22] that was used for forming through-silicon vias (TSVs), the molten ZnAl alloy (with the low melting point as 380 °C) is fully filled into the molds of the whole wafer within 10 min. After cooling, demolding and saw-dicing, the chip-based 3D double-deck metal solenoid/channel structure is fabricated. By assembling a sliding permanent magnet bar into a hollowed channel inside the core of the solenoid, which is formed during the micromachining process for the silicon mold, relative movement between the solenoid and magnetic bar can occur under external vibration along the channel direction, thereby generating electricity with the chip-style double-deck solenoid EM harvester. In the following sections, we will describe design, fabrication and testing techniques of the developed EM harvester.

## 2. Design and Modeling

**Figure 1:**
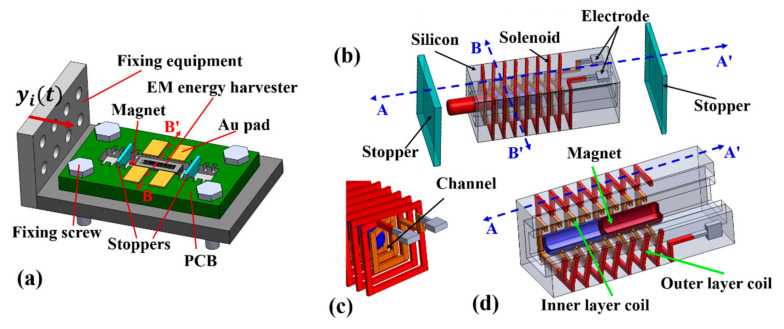
(**a**) Schematic prototype of the electromagnetic (EM) energy harvester; (**b**) Three-dimensional (3D) schematic of the chip-style harvester; (**c**) schematic of the double-deck solenoid; (**d**) cross section cut along the line of A-A’ in (**b**).

The experimental prototype of the EM energy harvester, based on impact/free motion [23], is schematically shown in Figure 1a, which mainly consists of the double-deck 3D metal solenoids EM energy harvester, two stoppers to limit the moving stroke of the sliding magnet and fixing equipment. A cylindrical permanent magnet is inserted into the central channel of the solenoid (see Figure 1b) that was pre-formed in the silicon substrate with the MEMS technique. When the amplitude of ambient vibration $y_{i}\left(t\right)$ along the channel direction is large enough, the permanent magnet can move freely and then collide with two stoppers. Due to the reciprocating relative-movement between the magnet and the solenoid, electrical power is generated in the solenoid by electromagnetic induction.

As shown in Figure 1b, the chip-style EM energy harvester consists of double-deck 3D metal solenoids, a cylindrical permanent magnet and two stoppers. The length of the commercial permanent magnet (NdFeB N53) is 10 mm and the diameter is 1 mm. The silicon mold for micro-casting the metal solenoid and the central channel to accommodate the sliding magnet (see Figure 1c) is fabricated in six silicon wafers (4-inch) that are further stacked together. The 3D solenoid-coil has to intertwine inside the six silicon layers. The inner layer coil (in yellow color) and the outer layer coil (in red) are clearly shown in Figure 1d. The metal coil of the inner solenoid and that of the outer solenoid are electrically connected in series, which move together relative to the sliding magnet. This configuration can greatly improve the power generating density of the chip-style energy harvester.

(1) $$M\ddot{y}+\left(C_{em}+C_{a}\right)\dot{x}+\frac{x}{\left|x\right|}F_{N}+\frac{\dot{x}}{\left|\dot{x}\right|}\mu Mg=0$$

When input vibration $y_{i}\left(t\right)$ is applied to the system, the equation of permanent magnet motion can be expressed as [23]:

Where M denotes the magnet mass, $x=y-y_{i}$ is the relative displacement between magnet and solenoid, y is the absolute magnet displacement,$\;C_{em}+C_{a}\;$represents the total viscous damping coefficient including electrical damping coefficient ($C_{em}$) and parasitic damping coefficient ($C_{a}$) [7], $F_{N}\;$is the impact force between magnet and stoppers, μ is coulomb’s friction coefficient, g is acceleration of gravity.

The contact between the magnet and the stopper can be approximately a cylindrical/half-space contact. Considering the elastic and damping characteristics of the colliding bodies, the impact force can be described as [24]:

(2) $$F_{N}=\left\{\begin{array}{c} k\theta^{n}+D\dot{\theta }\;\;\;\;\;\;\;\;\theta >0\\ 0\;\;\;\;\;\;\;\;\;\;\;\;\;\;\;\;\;\;\;\;\;\;\;\;\theta <0 \end{array}\right.\;$$

(3) $$\theta =\left|x\right|-s/2\;$$

where $k\theta^{n}$ and $D\dot{\theta }$ are elastic force and energy dissipation, respectively; k and D denote generalized stiffness parameters and hysteresis damping coefficient, respectively, θ is penetration in stopper, $\dot{\theta }$ represents relative penetration velocity, the exponent n is dependent on the contact surface (n=1 for cylinder/half-space contact) [25], s represents the magnetic stroke that is the full travelling distance of the magnet from one stopper to another.

For cylindrical/half-space contact, the generalized stiffness parameter k is given as [26]:

(4) $$k=\frac{2\cdot r_{\mathrm{mag}}}{3\cdot \left(\gamma_{m}+\gamma_{s}\right)}$$

(5) $$\gamma_{m}=\frac{1-v_{m}^{2}}{E_{m}},{\;\gamma }_{s}=\frac{1-v_{s}^{2}}{E_{s}}$$

where $r_{\mathrm{mag}}$ represents the magnet radius, $\gamma_{m}$ and $\gamma_{s}$ are material properties of magnet and stopper respectively, $E_{m}$ and $E_{s}$ are Young’s modulus of the magnet and stopper, respectively; $v_{m}$ and $v_{s}$ stand for Poisson’s ratio of magnet and stopper. The hysteresis damping coefficient D in Equation (2) is expressed by:

(6) $$D=\frac{3\left(1-e^{2}\right)\cdot k}{4\dot{\theta^{-}}}\theta^{n}$$

where e and $\dot{\theta^{-}}$ are restitution coefficient and penetration velocity just before impact.

In most linear vibration generators, the relative motion between the coil and the magnet is along one direction, e.g., assuming the direction is the x direction, and the magnetic field B is generated by one permanent magnet. Therefore, the induced voltage can be linked to the product of the flux linkage gradient and the velocity, which is given as [27]:

(7) $$U=-\frac{d\Phi }{dt}=-\frac{d\Phi }{dx}\;\frac{dx}{dt}=-\frac{d\Phi }{dx}\;\dot{x}$$

where Φ denotes total magnetic flux linkage through all the coil turns of the solenoid.

Power is output from the energy harvester by connecting the solenoid terminals with a load resistance to flow current to the loading resistor. This current creates a magnetic field which acts to oppose the permanent magnet field. The interaction between the induced field and the permanent magnet field generates the electromagnetic force that opposes the relative motion between magnet and solenoid. The instantaneous electromagnetic force $F_{em}$ is described as the product of velocity $\dot{x}$ and electromagnetic damping $C_{em}\;$[27].

(8) $$F_{em}\left(t\right)=C_{em}\cdot \;\dot{x}$$

In order to obtain the maximum electric power, it is important to design the energy harvester with maximum electromagnetic damping$\;C_{em}$. The instantaneous power extracted by the electromagnetic force can be expressed with the product of velocity$\;\dot{x}$ and force $F_{em}\;$as

(9) $$P_{e}=F_{em}\left(t\right)\cdot \;\dot{x}$$

The instantaneous power is dissipated in the load impedance and coil, which can be given by:

(10) $$P_{e}=\frac{U^{2}}{R_{\mathrm{coil}}+R_{\mathrm{load}}+j\omega L_{\mathrm{coil}}}$$

where $R_{\mathrm{coil}}$ is coil resistance, $R_{\mathrm{load}}$ denotes load resistance, $L_{\mathrm{coil}}$ represents coil inductance, ω=2πf is angular frequency of excitation, f is the frequency of excitation.

Therefore, the electromagnetic damping is

(11) $$C_{em}=\frac{1}{R_{\mathrm{coil}}+R_{\mathrm{load}}+j\omega L_{\mathrm{coil}}}\;\cdot {\left(\frac{d\Phi }{dx}\right)}^{2}$$

The energy conversion efficiency η of the energy harvester is given by:

(12) $$\eta =\frac{E_{\mathrm{electrical}}}{E_{\mathrm{input}}}$$

where $E_{\mathrm{electrical}}$ represents output electrical energy and $E_{\mathrm{input}}\;$denotes input vibration energy.

The analytic model for this novel EM energy harvester has been described as a numerically solved approach. The mechanical parameters, material and geometry parameters are designed based on the model prediction and listed in Table 1 and Table 2.

**Figure 2:**
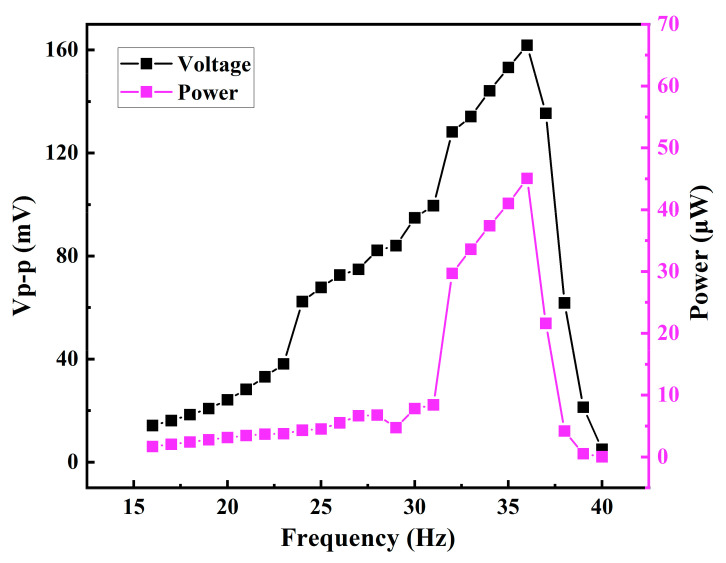
Simulated harvesting performance: peak-to-peak voltage and averaged power output in terms of frequency.

Based on the design, the performance of the harvester is analyzed, with the obtained prediction results shown in Figure 2. When the vibration frequency is less than 36 Hz, the peak-to-peak voltage and averaged output power both keep increase with the increasing vibration frequency. As for the vibration frequency to be higher than 36 Hz, the output voltage and power drop rapidly. The results show that the maximum average power reaches 45.07 μW at 36 Hz.

## 3. Fabrication

Our group recently developed a MEMS fabrication technique to fill molten metal into pre-fabricated micro-mold in silicon wafers to form a 3D metal solenoid which contains many turns of dense coils [28]. We named the technique micro-casting and it can be used to form on-chip integrated fluxgate sensors or transformers. After fulfilling the low melting-point metal into the silicon mold, the liquid metal outside the solenoid structure can be pinched off at the filling nozzles, due to the fact that the micromachined nozzles in a pre-fabricated silicon nozzle wafer were designed in a slim shape with a aspect ratio higher than two. Then the solenoid wafer is cooled down to room temperature and demolded from the top-cover wafer and the bottom nozzle wafer. In this way, solenoid wafers can be sequentially fabricated one by one and the nozzle wafer and the top-cover wafer can be repeatedly used. The technical details of the micro-casting equipment and the fabrication processes including the mold wafer preparation by using deep reactive ion etching (DRIE) and the molten metal filling procedure can be referred to in [28].

**Figure 3:**
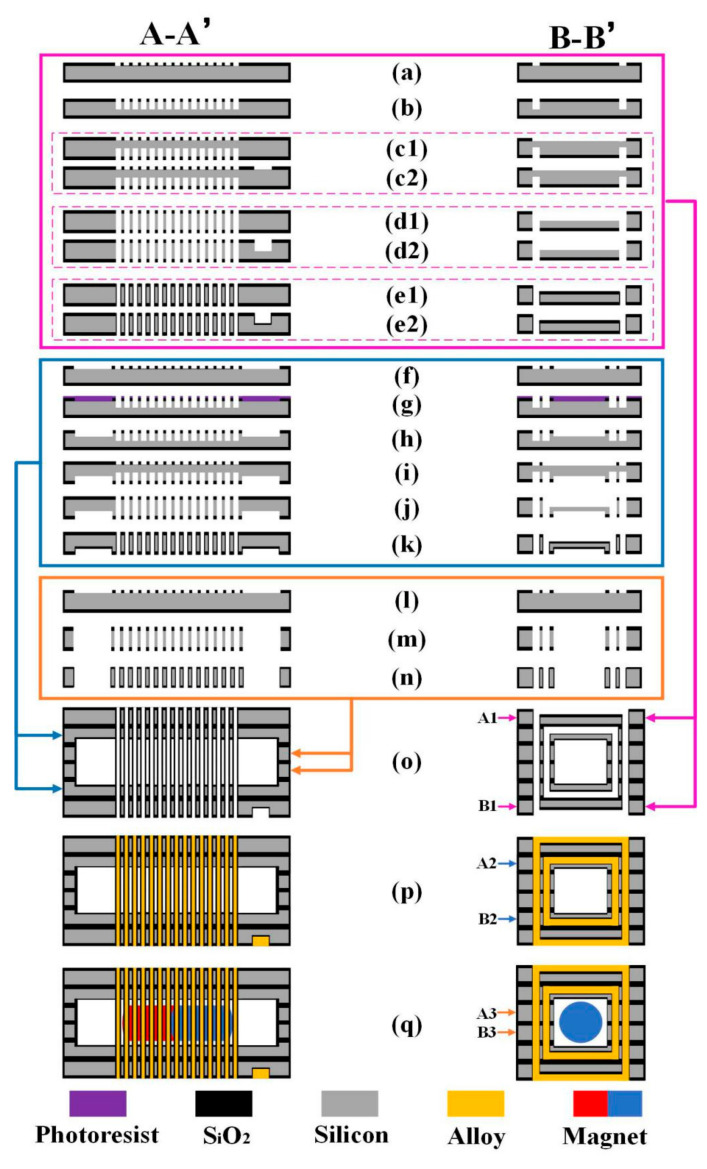
Fabrication process of the silicon-chip based solenoid. The (A-A’) and (B-B’) cross sections are defined in Figure 1b. (**a**–**e2**) Formation of the grooves and vias of the outer-layer coil (for the silicon mold layers of A1 and B1); (**f**,**g**) formation of the vias of inner-layer coil (for A2 and B2); (**h**) etching to form the channel for the sliding magnet; (**i**,**j**) etching to form the inner-layer-coil grooves; (**k**) oxidation to insulate the surface mold; (**l**–**n**) etching and oxidation for the middle support layers (for A3 and B3); (**o**) stacking the six silicon layers to form the whole mold for metal casting; (**p**) formation of the solenoid by ZnAl-alloy casting and cooling; (**q**) inserting cylindrical magnet.

To form this double-deck solenoid, we use ZnAl alloy as the filling metal with the melting point as 380 °C. After cooling, the alloy features about 3.6 times the resistivity of copper. The step-by-step fabrication process for the double-deck solenoid is shown in Figure 3 and described as follows. Please refer to Figure 3o–q, the silicon mold layers are numbered from top to bottom as A1, A2, A3, B3, B2 and B1. The steps from a to e2 are for A1 and B1 silicon layers, from f to k are for A2 and B2 silicon layers, from *l* to *n* are for A3 and B3 silicon layers. The A-A’ and B-B’ cross sections in Figure 3 can be referred to as in the indication in Figure 1b.

(a) 2 μm-thick SiO_2_ is thermally grown and patterned to form etching windows for the vias of the outer-layer top-coil in the A1 silicon mold and the outer-layer bottom-coil in the B1 silicon mold.

(b) The vias are etched by using the DRIE technique.

(c1) The 2 μm-thick SiO_2_ on the back of the silicon wafer is patterned to form the etching windows for the grooves of A1 silicon mold.

(c2) The SiO_2_ on the back of the silicon wafer is patterned to form the etching windows for the grooves and electrode of the B1 silicon mold.

(d1) The grooves in the A1 silicon mold are etched by using DRIE technique. Meanwhile the through-silicon vias in the A1 silicon mold are also etched through.

(d2) The grooves and electrode in the B1 silicon mold are etched by using the DRIE technique. At the same time, the through-silicon vias of the B1 silicon mold are also etched through.

(e1)–(e2) The A1 silicon mold and B1 silicon mold are oxidized to secure electric isolation between the metal wires and the silicon substrate for the outer-layer coil. The SiO_2_ thickness is 1 μm.

(f) The SiO_2_ is patterned to form the etching windows for vias of the inner-layer top-coil in the A2 mold and the inner-layer bottom-coil in the B2 mold.

(g) With the photoresist as mask, the vias of the A2 silicon mold and the B2 silicon mold are etched by using DRIE technique.

(h) The residual photoresist is moved away. Then, the cavity for the magnet sliding channel and the vias of the A2 silicon mold and B2 silicon mold are etched simultaneously with the DRIE technique.

(i) The SiO_2_ at the backside of A2 silicon mold and B2 silicon mold are patterned and etched to form the windows for the following groove etching.

(j) The grooves of the A2 silicon mold and B2 silicon mold are etched by using the DRIE technique. The through-silicon vias are also etched through.

(k) The A2 silicon mold and B2 silicon mold are oxidized to secure isolation between the metal wires and the silicon molds for inner-layer coil. The SiO_2_ thickness is 1 μm.

(l) The SiO_2_ is patterned to form the etch windows for the vias of the middle support layers of A3 and B3.

(m) Through-silicon vias are etched through with the DRIE technique.

(n) The A3 silicon mold and B3 silicon mold are oxidized to secure the insulation between the metal wires and the middle-support silicon molds.

(o) The six silicon molds are aligned and stacked together to form an assembled silicon mold. The channel for the magnet sliding is located at the center of the assembled silicon mold.

(p) The grooves and the vias formed in the stacked silicon mold are filled with the molten ZnAl alloy. After cooling and solidification, the complete 3D double-deck solenoids are formed.

(q) A cylindrical NeFeB permanent magnet is inserted into the channel.

**Figure 4:**
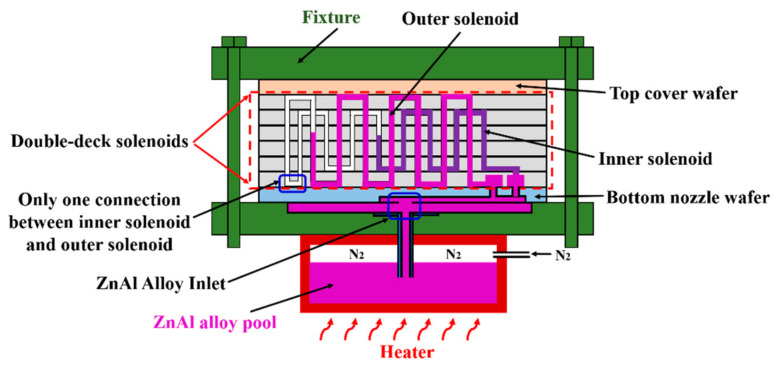
Schematic of the molten metal casting setup. At the moment the double-deck solenoid has been partly filled. The details of the casting process, including the following cooling, solidification and the casted wafer demolding from the top-cover and the bottom nozzle wafers, can be read in reference [28].

At the casting step (p) where the ZnAl alloy is filled, the technical details can be referred to our previously published literature [28]. For a better understanding, a schematic of the metal casting setup is shown in Figure 4 where both the inner-deck and outer-deck coils are partly filled with ZnAl alloy. The assembly mold is placed in the micro-casting equipment. The metal filling nozzles previously fabricated in the nozzle silicon wafer is aligned to the metal inlet vias of the model. Pressured by nitrogen gas, the molten ZnAl alloy flows out of the pool and inject into the solenoid mold through the nozzle and the casting process can be implemented at a wafer level. After several minutes, the solenoid mold can be fully filled with the ZnAl alloy. After cooling to room temperature, the wafer containing the formed solenoids can be demolded from the top cover wafer and the bottom nozzle wafer. And the metal casting process can be repeatedly implemented for the next silicon mold wafer.

**Figure 5:**
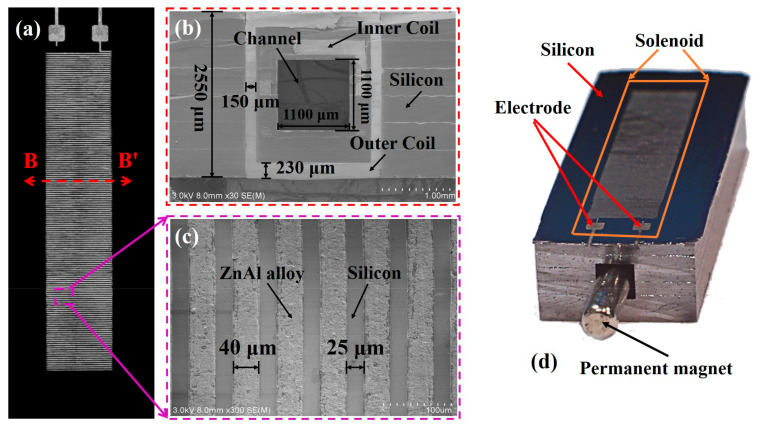
Fabricated double-deck 3D metal solenoid. (**a**) Top view scanning electron microscope (SEM) image of the solenoid. (**b**) Cross- sectional view of the solenoids that is cut along the line B-B’ in (**a**); (**c**) enlarged top view showing the ZnAl-alloy wires; (**d**) photograph.

The fabricated double-deck 3D solenoid is shown in Figure 5. The scanning electron microscope (SEM) image in Figure 5a shows the top view of the solenoid, where only the 150 turns of outer coil can be seen. Figure 5b shows the cross-sectional view of the solenoid, where both the outer coil metal and the inner coil metal are demonstrated. Figure 5c is the close-up view of the metal lines in Figure 5a. The 300-turn double-deck metal solenoid occupies a space of 10.58 mm ×2.06 mm ×2.55 mm. The number of turns of either the inner or the outer coil is 150 turns. As shown in Figure 5b, a total of six silicon-wafer layers are surrounded and tied by the 3D double-deck metal solenoid. The inner solenoid wounds around four layers of silicon substrate and the outer solenoid interleaves six layers of silicon. The cross-sectional dimensions of the channel for accommodating the sliding magnet is 1.1 mm×1.1 mm. As shown in Figure 5b,c, the thickness of the ZnAl-alloy coil is 230 μm for the lateral wires and 150 μm for the vertical wires, as well as, the metal width it is 40 μm with a space of 25 μm. Figure 5d is the photograph of the solenoid-containing silicon chip that is saw-diced from a 4-inch wafer and with the magnet inserted.

## 4. Testing Results

**Figure 6:**
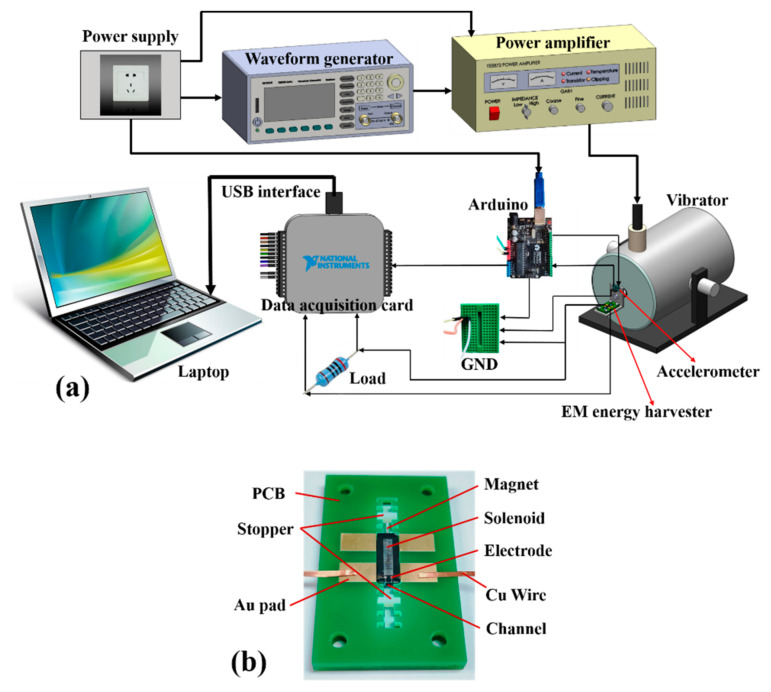
Schematic of the testing system. (**a**) Testing apparatus; (**b**) energy harvester prototype for test.

Two kinds of motion platform are designed for assessing the performance of the EM energy harvester prototype. The first one is a single direction vibration testing setup, where the excitation source is continuous sinusoidal mechanical vibration. The testing setup is schematically shown in Figure 6a. The prototype EM energy harvester is mounted on a board (shown in Figure 6b) and fixed onto a commercial JZK-5 shaker. A waveform generator (Agilent 33120A, Agilent Technologies, Santa Clara, CA, USA) is used to excite the shaker together with the harvester. The shaking direction is consistent with the magnet sliding direction. With the combination of waveform generator, power amplifier and shaker, the vibration is generated to excite the energy harvester. A standard accelerometer is fixed on the harvester prototype to sense the vibrating acceleration. A data acquisition card (NI USB-6003, 16bit, Engineer Ambitiously, Austin, TX, USA) is used to collect the voltage generated from the energy harvester. The sampled data of voltage and acceleration is transmitted into a laptop through a USB interface. The acceleration signal contains both the vibration amplitude and the frequency. By adjusting the acceleration frequency and amplitude, different vibration conditions for the energy harvester are obtained. In this way, the experimental results can be acquired under different vibratory accelerations.

The second method is wearable testing. The excitation coming from human-body motion is discontinuous sway. The EM kinetic energy harvester and a standard accelerometer are attached on the wrist of human-body. The wearable testing is carried out under three different types of human-body motions of walking, running and jumping. Accordingly, the three different experimental results are obtained.

**Figure 7:**
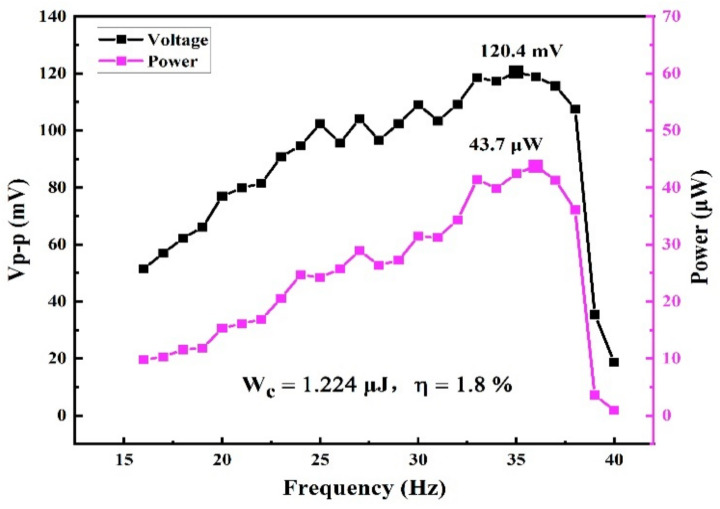
Experimental results of peak-to-peak voltage and average power output.

Firstly, the chip-style EM energy harvester is tested under continuous sinusoidal vibration. Giving different vibration amplitudes and frequencies, the EM energy harvester is excited and the relative motion occurs between the cylindrical permanent magnet and the solenoidal coil. By changing vibration frequencies from 16 Hz to 40 Hz, the experimental results of the peak-to-peak voltage and the average power output are shown in Figure 7. The maximum peak-to-peak voltage and average power output are obtained, and the tested results are 120.4 mV and 43.7 μW respectively. Compared with the simulated results shown in Figure 2, the power generation performance of the tested device agrees well with the designed result, thereby verifying the validity of the design model. The experimental result is a little bit lower than in the design. The reason for this lies in the fact that: (a) some inevitable defects existed in the solenoids coil result in larger internal resistance of the solenoid, thereby leading to more ohmic loss of coil resistance; (b) the energy loss because of the magnet-stoppers impacting is not included in the design model. The energy conversion efficiency η is calculated under 8 g vibration at 36 Hz. When 8 g vibrating acceleration is applied to the EM energy harvester, the input vibration energy is calculated as 67.88 μJ and the generated electrical energy within one vibration period is 1.224 μJ. Therefore, the energy conversion efficiency is calculated as 1.8%.

**Figure 8:**
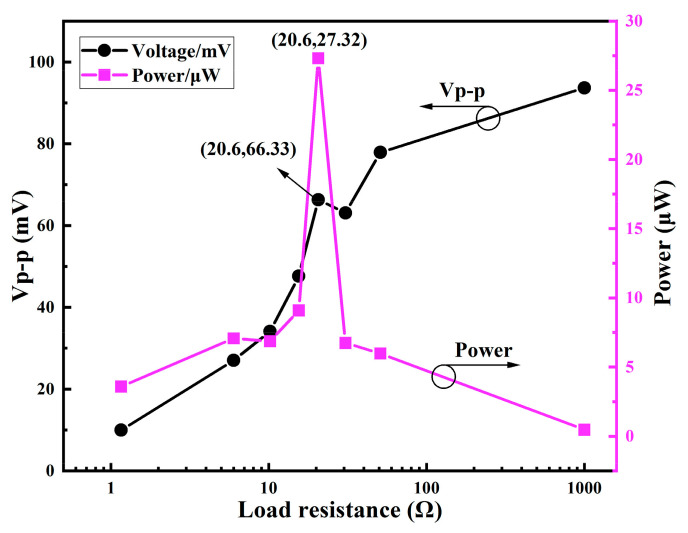
Peak-to-peak voltage and averaged power under different load resistances at 36 Hz.

Figure 8 shows the tested electric-generation results when the EM energy harvester is loaded with different resistances. The tested loading resistance is ranged from 1 Ω to 1000 Ω. In addition, the vibration frequency and the acceleration amplitude are fixed at 36 Hz and 8 g, respectively. The direction of the sinusoidal vibration is along the magnet sliding direction. When the load resistance is matched with the resistance of the solenoid itself, the maximum average power is output. The maximum average power output is 27.32 μW when the load resistance is 20.6 Ω. The measured resistor of the ZnAl-alloy solenoid is 17.9 Ω, which is a little bit lower than this matching value of 20.6 Ω. The reason probably lies in the fact that the bonded wires for leading out the signal and the interconnection line in the testing set up contribute extra resistance that should be attributed to the coil resistance.

**Figure 9:**
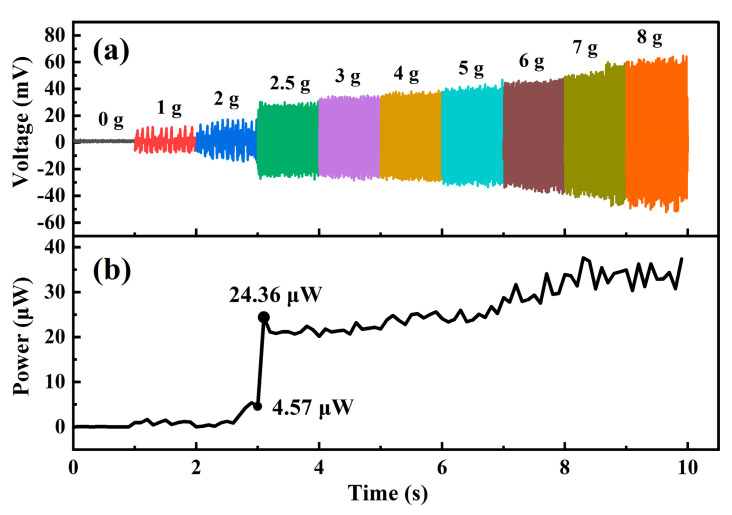
(**a**) Measured transient voltage, and (**b**) averaged power output under different acceleration amplitudes ranged from 0 g to 8 g, where the frequency is fixed as 36 Hz.

Figure 9 shows the threshold-triggering property of the EM kinetic energy harvester. The frequency of the sinusoidal mechanical vibration is fixed at 36 Hz. By controlling the amplitude of the vibrational displacement, the energy harvester can vibrate under different acceleration amplitudes, and the corresponding voltage and average power output are obtained.

Figure 9a shows the transient voltage generated from the energy harvester. In general, the generated voltage increases along with increasing the vibration-acceleration amplitude. The corresponding average power versus vibration acceleration is shown in Figure 9b. While the vibration acceleration reaches a threshold acceleration of about 2.5 g, the average power increases sharply. The reason is that the impact between magnet and stoppers occurs. At the impact moment the moving velocity of the magnet has a great change, thereby the magnetic flux linkage gradient $\frac{d\Phi }{dx}\cdot \frac{dx}{dt}$ changes sharply and contributes to the step increase in average power. After the vibration acceleration rises higher than the threshold, the generated voltage and average power output increase gradually by increasing the vibration acceleration. Figure 9b shows the triggering phenomenon of the energy harvester under 36 Hz. After the triggering occurred at about 2.5 g, the generated average power is suddenly lifted to 24.36 μW. It is worth indicating that, the threshold triggering phenomenon has potential to be used as a sensor switch for self-powered vibration monitoring applications. We will investigate it in depth in our future work.

**Figure 10:**
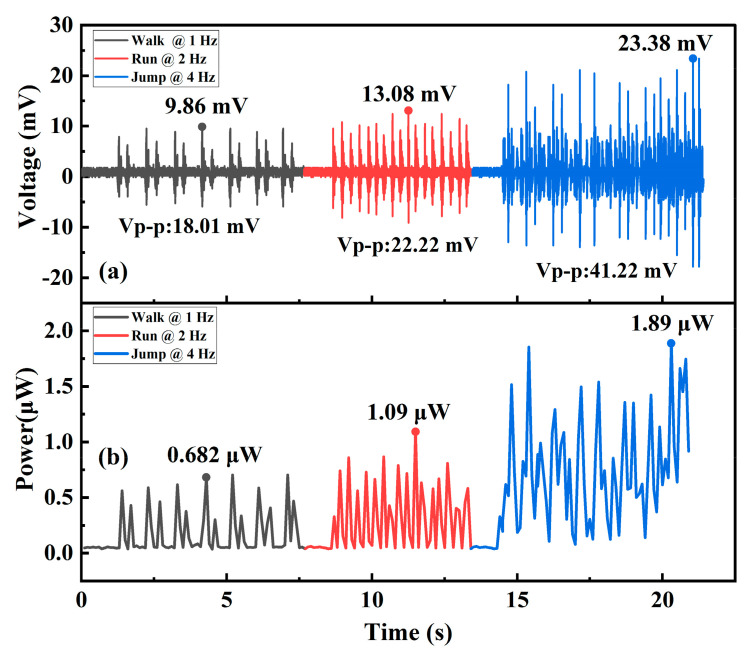
Wearable testing results for generated voltage and average power under different human-body movement modes. (**a**) Generated voltage under walking, running and jumping, sequentially from left to right; (**b**) consequent average power under human walking, running and jumping.

For potential wearable applications, the device was fixed on the wrist to be excited by human body movement. Then the EM energy harvester was tested, with the results shown in Figure 10. Electric voltage and power were generated under different human motions of walking, running and jumping. The peak-to-peak voltage and the average power during walking were 18.01 mV and 0.682 μW at about 1 Hz. Under running at about 2 Hz, the peak-to-peak voltage and the average power were 22.22 mV and 1.09 μW. The peak-to-peak voltage and average power during repeated jumps (at about 4 Hz) reached 41.22 mV and 1.89 μW. The small sized chip-style EM energy harvester is promising for human motion pattern recognition or even for a power supply for wearable microsystems. The device can be imbedded into wearable electrical devices to convert human-body kinetic energy into electrical energy for self-powering wearable applications. Considering that the solid magnet bar should slide in the channel, the chip substrate cannot be in a flexible shape.

The overall performance of the proposed kinetic energy harvester has been compared with that of previously reported kinetic energy harvesters. Table 3 compares the performance of the harvester in this work to those reported energy harvesters with different power generation mechanisms: the electromagnetic energy harvester (EMEH), piezoelectric energy harvester (PEEH), piezo-electromagnetic energy harvester (PEMEH), electrostatic energy harvester (ESEH), thermoelectric generator (TEG), triboelectric nanogenerator (TENG), and pyroelectric generator (PyG), and heat radiation pyroelectric generator (HRPyG). The piezo-electromagnetic energy harvester can generate relatively high power, but its power density is still not very high due to its very large size. By overall consideration of power generation and device volume, the power density and power density per unit gravity acceleration of our proposed chip-style EM energy harvester are both the highest. Our proposed harvester has greatly improved the power density per unit gravity acceleration, which is almost 31 times higher than that of the published ones under low-frequency (less than 100 Hz) vibration. Furthermore, the output current is even 3180 times higher than that of the published harvesters, thanks to the low internal resistance and relatively high power generation of this work.

## 5. Conclusions

This study proposes a novel double-deck 3D metal solenoid integrated in a silicon wafer for a low-frequency EM kinetic energy harvester. The measured maximum peak-to-peak voltage and average power output were 120.4 mV and 43.7 μW, which occurred under 8 g ambient vibration at 36 Hz. Furthermore, the energy harvester showed a threshold-triggering property. While the measured vibration acceleration reached the threshold acceleration of about 2.5 g, the average power experienced a sudden step-increase. The proposed EM energy harvester can also generate electricity under different kinds of human body movement: walking, running and jumping, where the average power was 0.682 μW, 1.09 μW and 1.89 μW, respectively. Due to its high efficiency power generation performance and threshold-triggered property, this proposed EM kinetic energy harvester with chip-style 3D double-deck metal solenoid structure has the prospect to sustain a low-power sensor or sensing switch for self-powered vibration monitoring applications like human motion pattern recognition or wearable microsystems.

## Figures and Tables

**Table 1:** Designed mechanical parameters of the solenoid.

Parameter	Symbol	Value
Young’s modulus of magnet	$E_{m}\;\left(\mathrm{GPa}\right)$	160
Young’s modulus of stopper	$E_{s}\;\left(\mathrm{GPa}\right)$	1
Cylindrical magnet diameter	$d_{m\mathrm{ag}}\;\left(\mathrm{mm}\right)$	1
Cylindrical magnet length	$l_{m\mathrm{ag}}\left(\mathrm{mm}\right)$	10
Density of cylindrical magnet	$\rho \;\left(\mathrm{kg}/m^{3}\right)$	7500
Magnet stroke	$s\;\left(\mathrm{mm}\right)$	11.4
Viscous coefficient	$C_{a}\;\left(\mathrm{Ns}/m\right)$	0.01
Poisson’s ratio of magnet	$v_{m}$	0.24
Poisson’s ratio of stopper	$v_{s}$	0.38
Restitution coefficient	e	0.5
Coulomb’s coefficient	μ	0.25
Solenoid length	$l\;\left(\mathrm{mm}\right)$	9.725

**Table 2:** Material and geometric parameters of the solenoid.

Parameter	Symbol	Value
Effective length of the device chip	$l_{s}\;\left(\mathrm{mm}\right)$	10.58
Effective width of the device chip	$w_{s}\;\left(\mathrm{mm}\right)$	2.06
Effective thickness of the device chip	$h_{s}\;\left(\mathrm{mm}\right)$	2.55
Width of the square-sectional channel	$w_{c}\;\left(\mathrm{mm}\right)$	1.1
Length of the square-sectional channel	$l_{c}\;\left(\mathrm{mm}\right)$	1.1
Wire width of the metal coil	$w_{\mathrm{coil}}\;\left(\mu m\right)$	40
Wire thickness of the metal coil	$t_{\mathrm{coil}}\;\left(\mu m\right)$	230
Gap distance between adjacent metal wires	$d_{\mathrm{coil}}\;\left(\mu m\right)$	25
ZnAl alloy resistivity	$\rho \left(\mu \Omega \cdot \mathrm{cm}\right)$	6.3
Number of the solenoid layers	$N_{l}$	2
Turns of each coil layer	$N_{t}$	150
Total turns of solenoid	N	2×150

**Table 3:** Power performance comparison with some recently published energy harvesters.

Reference	PGM ^1^	MAP ^2^ (μW)	FMP ^3^ (Hz)	Ambient Excitation	Device Volume $\left(cm^{3}\right)$	Resistance	Output Voltage $\left(mV\right)$	Current	PD ^4^ $\left(\frac{\mu W}{cm^{3}}\right)$	NPD ^5^ $\left(\frac{\mu W}{cm^{3}\cdot g}\right)$
This work	EMEH	43.7	36	8 g	0.0556	17.9 Ω	$120.4_{\left(\mathrm{pp}\right)}$	1.6 mA	786	98.3
[8]	PEEH	0.0855	36	1 g	0.016	330 MΩ	$75.5–115.5_{\left(\mathrm{rms}\right)}$	0.51 μA	5.34	5.34
[9]	PEMEH	1310	4.8	0.8 g	115.42	100 MΩ	$10150_{\left(\mathrm{pp}\right)}$	0.11 mA	11.35	14.2
[10]	ESEH	45	20	1.3 g	11.25	100 MΩ	-	21 μA	4	3.08
[12]	EMEH	12.7	4	2 g	1.1	80 Ω	6$1–93.7_{\left(\mathrm{rms}\right)}$	0.40 mA	11.5	5.75
[13]	EMEH	16.1	37	6.5 g	0.045	7.8 Ω	$37.05_{\left(\mathrm{pp}\right)}$	1.4 mA	357.8	55.1
[16]	TEG	290	-	ΔT:3.7–5.7 K	Area = 13.8 ${\mathrm{mm}}^{2}$	174–255 Ω	<$0.55_{\mathrm{means}}$	1.07 mA	0.115 $\mu W/{\mathrm{cm}}^{2}$	-
[17]	TENG	2.5 × 10^5^	7.5	Force = 20 N	Area = 48${\;\mathrm{cm}}^{2}$	5 MΩ	2,600,000	0.48 mA	5.23 × 10^3^ $\mu W/{\mathrm{cm}}^{2}$	-
[18]	PyG	0.0238	-	ΔT=30 K	Area = 7${\;\mathrm{cm}}^{2}$	1.5 MΩ	1500	1.5 μA	0.034 $\mu W/{\mathrm{cm}}^{2}$	-
[19]	PyG	8.31	-	ΔT=5 ℃	Area = 12.25${\;\mathrm{cm}}^{2}$	50 MΩ	42,000	2.5 μA	0.678 $\mu W/{\mathrm{cm}}^{2}$	-
[20]	HRPyG	0.76	0.5	*T* = 78 ℃	0.1	3 MΩ	$22,700_{\mathrm{pp}}$	0.503 μA	7.6	-
[21]	HRPyG	7.16	-	λ=9.85–11.35 μm	Area = 3.23${\;\mathrm{cm}}^{2}$	-	413	1.49 $\mu A/{\mathrm{cm}}^{2}$	2.2 $\mu W/{\mathrm{cm}}^{2}$	-

Note: ^1^ Power Generation Mechanism; ^2^ Maximum Average Power; ^3^ Frequency at Maximum Power; ^4^ Power Density; ^5^ Normalized Power Density.
